# The Clinical Efficacy of Fibrinogen Concentrate in Massive Obstetric Haemorrhage with Hypofibrinogenaemia

**DOI:** 10.1038/srep46749

**Published:** 2017-04-24

**Authors:** Shigetaka Matsunaga, Yasushi Takai, Eishin Nakamura, Sumiko Era, Yoshihisa Ono, Koji Yamamoto, Hiroo Maeda, Hiroyuki Seki

**Affiliations:** 1 Center for Maternal, Foetal and Neonatal Medicine, Saitama Medical Centre, Saitama Medical University, Kawagoe, Japan; 2 Department of Transfusion Medicine and Cell Therapy, Saitama Medical Centre, Saitama Medical University, Kawagoe, Japan

## Abstract

Massive obstetric haemorrhage remains a major cause of maternal death attributable to hypofibrinogenaemia. Transfusion of large volumes of fresh frozen plasma (FFP) is required to normalise fibrinogen levels. We compared the efficacy of FFP (F group) with that of FFP plus fibrinogen concentrate (F + F group) in massive obstetric haemorrhage. In this retrospective study, we compared the medical charts (2004–2016) of 137 patients with <150 mg/dl fibrinogen treated with F + F (n = 47; after August 2009) or F (n = 56; before August 2009). Although fibrinogen concentrate was only administered in severe cases, the FFP/red blood cell concentrate (RCC) ratio was significantly lower in the F + F group than in the F group. A sub-group analysis of cases requiring ≥18 RCC units showed that the F + F group received significantly less FFP than the F group (40.2 ± 19.6 versus 53.4 ± 18.5 units; *P* = 0.047) and showed significantly less pulmonary oedema (24.0% vs 57.1%; P < 0.05) in the absence of any significant differences in pre-transfusion coagulation, estimated blood loss, or RCC transfusion volume. Administration of fibrinogen concentrate increased the rate of fibrinogen supplementation five-fold and reduced FFP dosage, the FFP/RCC ratio, and the incidence of pulmonary oedema.

Massive obstetric haemorrhage remains a major cause of maternal mortality worldwide, currently accounting for 27.1% of all maternal deaths^1^. The pathology, although difficult to categorise clearly, can be subdivided into dilutional coagulopathy (attributable to causes such as genital tract trauma and atonic bleeding) and consumption coagulopathy (attributable to placental abruption or amniotic fluid embolism); both of these pathologies frequently result in disseminated intravascular coagulation, which is associated with extreme hypofibrinogenaemia^2^,^3^. We previously reported that the fibrinogen level at the time when the decision to perform blood transfusion was made correlated with the transfusion dosage^4^ and that fibrinogen level is the most sensitive determinant of the transfusion volume employed to treat massive obstetric haemorrhage^5^.

Rapid normalisation of fibrinogen levels reduces blood loss^6^. Currently, coagulation factors are supplemented using fresh frozen plasma (FFP) as the first-line therapy because it is widely available and is indicated for coagulopathy treatments in many countries. However, only a low level of fibrinogen is present in FFP^7^; we have previously reported that when FFP alone is used for coagulation factor supplementation in massive obstetric haemorrhage, the ratio of FFP to red blood cell concentrate (RCC) (FFP/RCC ratio) must be at least 1.0, regardless of the pathology underlying the haemorrhage^4^. Therefore, sufficient coagulation factor supplementation requires the transfusion of a large volume of FFP. Coagulation factor supplementation with FFP thus presents a number of problems. First, the use of a large volume of FFP results in a high rate of pulmonary oedema, associated with the increased circulating blood volume^5^. Second, FFP thawing requires manpower and its administration takes some time, thus slowing coagulation factor replenishment and prolonging coagulopathy; this can result in continued haemorrhage and subsequent transfusions^4^. Although FFP is the first-line agent used to treat acquired fibrinogen deficiencies in many countries, fibrinogen levels can be restored more rapidly by using fibrinogen concentrate or cryoprecipitate; in Japan, however, these are not covered by health insurance, and the required blood products are not widely available^8^. Fibrinogen concentrate can be used “off label” in acquired fibrinogen deficiencies and is also used as the standard treatment in some countries such as Austria, Germany, and Switzerland. Fibrinogen is an essential protein involved in platelet aggregation and blood coagulation. The use of fibrinogen and other coagulation factor concentrates that include high levels of fibrinogen should therefore provide therapeutic benefits by efficiently restoring the levels of key coagulation factors. However, there are few studies comparing the efficacy of these blood products in obstetric patients with that of a relevant control group^9^,^10^,^11^,^12^,^13^,^14^, and no reports have investigated this in a controlled clinical setting in individuals with hypofibrinogenaemia^15^ because these are generally emergency medicine patients. Therefore, in the present study we retrospectively compared the effects of administering a fibrinogen concentrate to patients treated for hypofibrinogenaemia (fibrinogen < 150 mg/dl) attributable to massive obstetric haemorrhage with those observed in patients treated with FFP alone, including reduced complications.

## Results

Table 1 shows patient demographics for the 103 patients involved in the present study who were treated with FFP and fibrinogen concentrate (F + F group, n = 47) or FFP alone (F group, n = 56). There were no significant differences in patient demographics between these two study groups.

Within the F + F group, we analysed the difference between the post- and pre-administration fibrinogen level (Δfibrinogen) and divided this by the duration of administration to calculate the rate of fibrinogen increase (Δfibrinogen/h) in 32 patients treated with 3 g fibrinogen concentrate and 15 patients who received 6 g fibrinogen concentrate (Table 2). Fibrinogen concentrate administration significantly increased fibrinogen levels (*P* < 0.0001). Those treated with 6 g of this preparation showed significantly higher Δfibrinogen and Δfibrinogen/h values than those treated with 3 g.

We also compared Δfibrinogen/h between the F + F and F groups (data not shown in table). This analysis found that the mean Δfibrinogen/h was significantly higher in the F + F group (123.0 ± 112.3) than in the F group (24.8 ± 19.3) (*P* < 0.0001).

**Figure 1:**
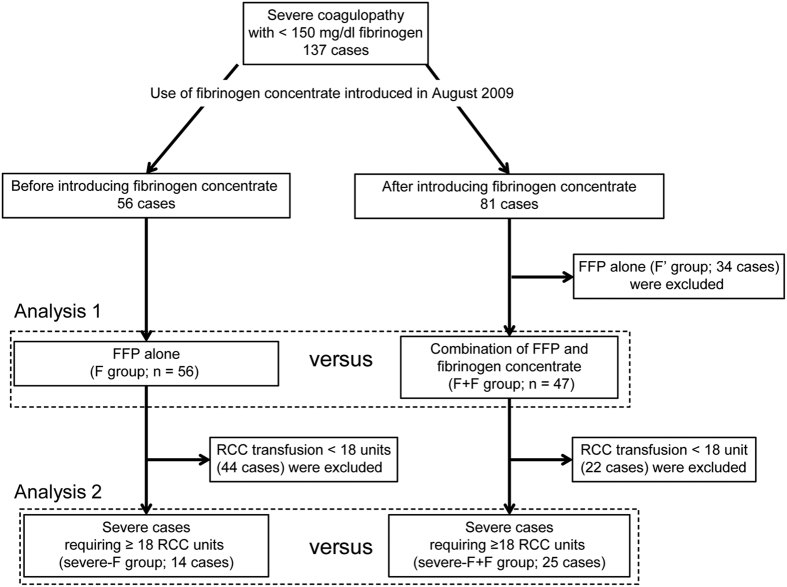
Flow chart showing the enrolled patients for this study.

In order to investigate the effects of fibrinogen concentrate, we analysed blood test data at the time of the decision to perform blood transfusion to determine the transfusion volume (Fig. 1, Analysis 1). Table 3 shows no significant differences in pre-transfusion blood fibrinogen levels in the F + F and F groups. The F + F group had a significantly lower prothrombin time activity percentage (PT%) and a significantly higher RCC dosage than that of the F group. Even though fibrinogen concentrate was predominantly administered in severe cases, the FFP/RCC ratio was significantly lower in the F + F group than in the F group. There was no significant difference in FFP dosage in these two groups. Thrombosis was not observed in any of the 47 patients in the F + F group.

**Figure 2:**
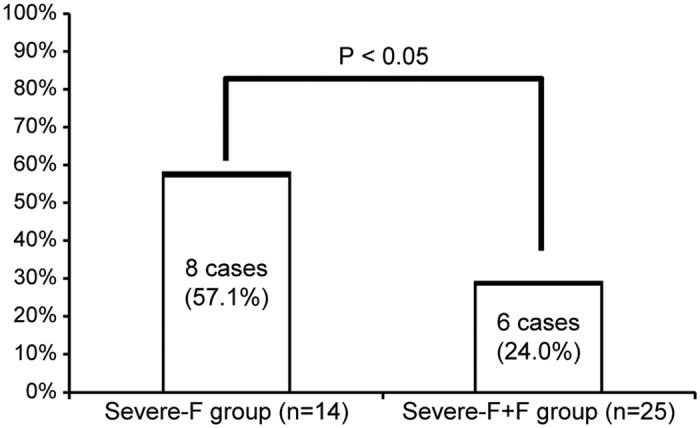
Incidence of pulmonary oedema in the most severe cases, receiving ≥18 red blood cell concentrate units. The severe-F group was treated with fresh frozen plasma and the severe-F + F group was treated with fresh frozen plasma and fibrinogen concentrate.

In addition, to match the severity of these groups, we performed sub-group analyses of the most severe cases (Fig. 1, Analysis 2). Although no significant differences were observed for pre-transfusion PT%, fibrinogen, estimated blood loss, or RCC dosage in these sub-groups requiring ≥ 18 RCC units, the severe-F + F group received a significantly lower dosage of FFP than that of the severe-F group (Table 3). Pulmonary oedema occurred in significantly fewer patients in the severe-F + F group than in the severe-F group (*P* < 0.05; Fig. 2).

We performed a statistical analysis between placental abruption and uterine atony in their early stages of severity. In patients with early-stage placental abruption requiring 10 units or less RCC, the F + F group required a significantly lower dosage of FFP and FFP/RCC than that of the F group, although RCC and estimated blood loss were not significantly different (Table 4). In contrast, in early-stage uterine atony cases requiring 10 units or less RCC, no significant differences were found between the F + F and F groups, although the number of cases was low (2 and 7 cases, respectively).

## Discussion

Compared to treatment using FFP alone, the combination of FFP and fibrinogen concentrate produced a more rapid increase in fibrinogen levels by roughly five-fold, reduced the FFP/RCC ratio, and allowed the administration of a lower dosage of FFP. Administration of fibrinogen concentrate also reduced the incidence of pulmonary oedema (a complication of FFP administration) and did not cause thrombosis in any of the patients included in this study. The results of the present study thus suggest that the use of FFP and fibrinogen concentrate in combination provides an effective treatment for massive obstetric haemorrhage.

As we and other researchers have previously reported, lower levels of fibrinogen require higher transfusion volumes of both RCC and FFP^2^,^5^. Therefore, rapid normalisation of the blood fibrinogen level by administration of fibrinogen concentrate is expected to reduce RCC transfusion volume and blood loss^16^. In the present study, the significantly higher RCC dosage observed in the F + F group (Table 3) may reflect the preferential use of fibrinogen concentrate in patients with more severe haemorrhages, which would be difficult to treat with FFP alone. Nevertheless, the FFP dosage necessary for coagulation factor supplementation was comparable between the two groups; thus, fibrinogen concentrate yielded a lower FFP/RCC ratio. In patients with equally severe haemorrhages, those in the F + F group would be expected to show a lower FFP/RCC ratio than those in the F group. Indeed, the present sub-group analysis of patients who received ≥18 RCC units shows that the severe-F + F group received not only a lower FFP/RCC dose, but also a significantly lower FFP dose than that of the severe-F group.

Although a few case series have been reported previously^17^, there are no controlled studies of the efficacy and complications of fibrinogen concentrate usage in patients with massive obstetric haemorrhage and hypofibrinogenaemia^9^,^10^,^11^,^12^,^13^,^14^,^15^. Only one study included a control group; however, this study compared the use of cryoprecipitate and fibrinogen concentrate rather that comparing the use and non-use of fibrinogen concentrate^18^. For this reason, it did not report any differences in the usage of transfusion products or blood loss. The present study suggests that the use of fibrinogen concentrate would be particularly effective for massive obstetric haemorrhage, although recent data relating to non-obstetric haemorrhage show no benefits for fibrinogen concentrate administration^19^. This may be because pregnancy enhances coagulation and reduces fibrinolysis^20^,^21^, which affects consumption of fibrinogen and hyperfibrinolysis in cases of massive obstetrical haemorrhage. In addition, the use of fibrinogen concentrate and FFP is the usual treatment method in many countries. Therefore, this study has novelty and is applicable to a realistic clinical situation.

Fibrinogen concentrate has a low risk of viral infection. Further, various antigens and antibodies that are present in cryoprecipitate have been removed. Fibrinogen concentrate can also be stored at room temperature. This means that no thawing is required, allowing for rapid restoration of the blood fibrinogen level to the target range to achieve haemostasis^22^.

As 600 ml FFP contains 1 g fibrinogen^23^, administration of 2 FFP units to hypofibrinogenaemic patients increases their blood fibrinogen level by 30–50 mg/dl. Thus, approximately 1,500 ml FFP is required to increase the fibrinogen level by 100 mg/dl. The use of FFP alone in patients with a massive obstetric haemorrhage requires an FFP volume large enough to achieve an FFP/RCC ratio >1.0 when converted to whole-blood ratio^4^. In contrast, administration of 1 g fibrinogen concentrate in 50 ml increases the blood fibrinogen level by 30 mg/dl and 3–4 g (150–200 ml) can therefore increase the fibrinogen level by 100 mg/dl^24^. Thus, the much smaller volume of fibrinogen concentrate required to correct coagulation factor levels reduces the incidence of pulmonary oedema, as shown in the present study. As mentioned above and in previous reports^10^,^13^,^17^,^25^, we chose 3 g as the fibrinogen concentrate dose.

Patients with extremely severe haemorrhages of >4000 ml require a large volume of fibrinogen supplementation, as well as treatment for the resultant dilutional coagulopathy and depletion of coagulation factors other than fibrinogen. Prior to beginning rapid coagulation factor supplementation using fibrinogen concentrate, infusion and RCC transfusion may further exacerbate dilutional coagulopathy, thus necessitating supplementation of these other coagulation factors. While the lowest fibrinogen level commensurate with haemostasis is 40–50% of the normal range (100 mg/dl), the minimum level of most other coagulation factors is 20–25% of their normal range^26^. This means that some massive haemorrhages can be treated by fibrinogen supplementation, while others require both fibrinogen and other coagulation factors^4^,^22^. The severe-F + F group consisted of patients who required ≥18 RCC units and was inferred to include many patients with severe dilutional coagulopathy, where coagulation factors besides fibrinogen were below the minimum levels required for haemostasis; the need for administration of coagulation factors besides fibrinogen was therefore high. Nevertheless, we found that FFP dosage was in fact greatly reduced in the severe-F + F group (Table 3).

In extremely severe haemorrhages of >4000 ml, continuous administration of fibrinogen concentrate (without dosage limits) and FFP may be warranted to maintain a fibrinogen level of ≥150 mg/dl, thus preventing further blood loss and reducing the transfusion volume. Future studies should investigate the use of cryoprecipitate and other blood products containing large amounts of coagulation factors other than fibrinogen; these have the potential to produce effective reduction of blood loss in dilutional coagulopathy and to reduce the dose of RCC.

In the early stages of placental abruption, a representative cause of consumption coagulopathy, the circulating blood volume is maintained; however, extrinsic coagulation factors are reduced, attributable to the influx of tissue thromboplastin into the maternal blood, while other coagulation factors are relatively preserved. Consequently, rapid fibrinogen supplementation immediately improves coagulopathy and achieves haemostasis. Therefore, as shown in Table 4, the use of fibrinogen concentrate would be very beneficial in the early stages of consumption coagulopathy caused by placental abruption because it would minimise blood loss and transfusion volume^21^,^27^.

The present study was limited by being a retrospective and relatively small epidemiological study. However, large-scale randomised controlled trials in this area are difficult because fibrinogen concentrate is not indicated for the treatment of massive obstetric haemorrhage in many countries, and it would be ethically problematic to withhold this treatment from the control group in countries where it is normally available. The efficacy of fibrinogen concentrate is demonstrated in very severe cases requiring a large RCC transfusion, as well as in early-stage placental abruption cases requiring a small RCC transfusion. An additional limitation is the absence of a group treated with fibrinogen concentrate alone. Administration of fibrinogen concentrate and fluids might be appropriate for the treatment of coagulopathy under certain circumstances. Because this study was conducted over a long period, learning bias might also affect the results. Although our study is retrospective, we would like to add more cases to it in the future to improve the precision of these analyses.

## Materials and Methods

This study was conducted in accordance with the approval of the Saitama Medical Center/Saitama Medical University Institutional Review Board. The requirement for informed consent was waived because this study was a retrospective medical chart review. We announced our study plan and advertised for opposition letters from patients on our Center’s website during the study period. All methods were performed in accordance with Ethical Guidelines for Medical and Health Research Involving Human Subjects issued by Ministry of Education, Culture, Sports, Science and Technology and Ministry of Health, Labour and Welfare of Japan (http://www.lifescience.mext.go.jp/files/pdf/n1500_01.pdf).

We identified 137 patients with severe coagulopathy attributable to massive obstetric haemorrhage between 2004 and 2016 who presented with a fibrinogen level <150 mg/dl and underwent transfusion therapy with FFP or FFP plus fibrinogen concentrate.

Fibrinogen levels were measured using a STA-R Evolution analyser (Roche Diagnostics K.K., Tokyo, Japan). Clinical decisions to transfuse were based on the “Our transfusion management principle” document, which was consistent from 2004 onwards^4^.

In Japan, 2 units of RCC/FFP are made from 400 ml whole blood.

The ultimate goal of RCC transfusion, based on a consideration of the oxygen-carrying capacity, was 7 g/dl haemoglobin^6^,^28^, while the final goals for FFP transfusion were a PT% of 60% and a fibrinogen level of 150 mg/dl^29^,^30^; this was defined as the point at which haemostasis was achieved. We performed repeated haemoglobin tests after administration of every 2–6 units of RCC at approximately the same time as the coagulation function test. RCC was administered until the final goals were reached and the administration of 2 RCC units increased haemoglobin levels by 1.6–1.7 g/dl. We performed repeated coagulation function tests after administration of every 6–8 units of FFP. FFP was administered until the aforementioned final goals were reached, and administration of 2 FFP units to hypofibrinogenaemic patients increased their blood fibrinogen levels by 30–50 mg/dl. We also noted RCC and FFP during transfusion, and maintained a FFP/RCC ratio above 1.0^4^ for every coagulation function test.

Fibrinogen concentrate was administered in addition to FFP as an aggressive coagulation factor supplementation. The initial dose employed was 3 g. The fibrinogen level was determined again 15 min after administration; if it remained below 150 mg/dl, a further 3 g was administered. The fibrinogen level was then tested again, and a maximum of 6 g fibrinogen concentrate was administered.

Uterotonic agents such as oxytocin and methylergometrine were routinely administered to patients with uterine atony.

Pulmonary oedema, associated with an increase in circulating blood volume, was defined as an arterial oxygen saturation as measured by pulse oximetry (SpO_2_) <95% with reduced chest X-ray permeability, requiring oxygenation and diuretic administration. Transfusion-related acute lung injury^31^, a serious blood transfusion reaction characterised by dyspnoea attributable to non-cardiogenic sudden pulmonary oedema within several hours of transfusion, was not observed in individuals with pulmonary oedema.

As shown in Fig. 1, we divided patients into two groups treated before or after the introduction of fibrinogen concentrate in August 2009. One group of patients was treated with F + F after August 2009 (n = 47), while those who received FFP transfusions prior to August 2009 were defined as the F group (n = 56). In general, fibrinogen concentrate is only administered in severe cases; therefore, the group of patients treated with FFP alone from August 2009 onwards (F’ group; n = 34) were mild cases in comparison to the F + F group; these patients were excluded from further analyses (Analysis 1). To match the severity of conditions between the study groups, we also conducted a sub-group analysis of the most severe cases, defined as those requiring ≥18 RCC units (Analysis 2).

Statistical analyses were performed using JMP v10.0 (SAS Institute, Cary, NC, USA). The study groups were compared using the Kruskal-Wallis test and chi-square test, with *P* < 0.05 considered to indicate a statistically significant difference.

## Additional Information

**How to cite this article:** Matsunaga, S. *et al*. The Clinical Efficacy of Fibrinogen Concentrate in Massive Obstetric Haemorrhage with Hypofibrinogenaemia. *Sci. Rep.* **7**, 46749; doi: 10.1038/srep46749 (2017).

**Publisher's note:** Springer Nature remains neutral with regard to jurisdictional claims in published maps and institutional affiliations.

## Figures and Tables

**Table 1:** Patient demographics.

	F + F group	F group	*P*-value
	Number (%)	Number (%)	
Pathogenesis			
Uterine atony	17 (36.2)	19 (33.9)	>0.05
Placental abruption	23 (48.9)	30 (53.6)	>0.05
Genital tract trauma	3 (6.4)	6 (10.7)	>0.05
Others	4 (8.5)	1 (1.8)	>0.05
** Total**	**47 (100)**	**56 (100)**	
Background			
Age	34 ± 4.4	32 ± 4.7	>0.05
History of MOH	2 (4.2)	1 (1.7)	>0.05
Gynaecologic complication	6 (12.7)	7 (12.5)	>0.05
Uterine fibroids	4 (8.5)	4 (7.1)	>0.05
Post myomectomy	2 (4.2)	0 (0.0)	>0.05
Uterine abnormality	0 (0.0)	1 (1.7)	>0.05
Endometriosis	0 (0.0)	1 (1.7)	>0.05
Post ovarian bleeding	0 (0.0)	1 (1.7)	>0.05
Haemostatic procedures			
Intrauterine gauze or balloon	22 (46.8)	34 (60.7)	>0.05
TAE	15 (31.9)	14 (25.0)	>0.05
Hysterectomy	4 (8.5)	1 (1.7)	>0.05
Internal iliac artery ligation	1 (2.1)	0 (0.0)	>0.05
Uterotonic agents			
Oxytocin	46 (97.8)	56 (100)	>0.05
Methylergometrine	35 (74.4)	44 (78.5)	>0.05

MOH, massive obstetric haemorrhage; TAE, transcatheter arterial embolization; F + F group, fresh frozen plasma and fibrinogen concentrate; F group, fresh frozen plasma alone.

**Table 2:** Blood fibrinogen levels in patients treated with the indicated doses of fibrinogen concentrate.

	Median	Mean ± SD	95% CI	n
Δfibrinogen (total)	118	132 ± 60.2	114.5–149.9	47
Δfibrinogen (3 g)	106.5	105.2 ± 39.1	91.1–119.3	32
Δfibrinogen (6 g)	214	189.8 ± 57.4	157.9–221.6	15
Δfibrinogen/h (total)	80.7	123.0 ± 112.3	90.1–156.0	47
Δfibrinogen/h (3 g)	71.4	93.9 ± 96.3	59.2–128.7	32
Δfibrinogen/h (6 g)	166	185.2 ± 121.8	117.7–252.7	15

Δfibrinogen, the difference between post- and pre-administration fibrinogen levels; Δfibrinogen/h, the Δfibrinogen divided by the time required for administration; SD, standard deviation; CI, confidence interval.

**Table 3:** Comparison of fibrinogen levels and other haemostatic parameters before treatment, and blood product usage in the groups treated with fresh frozen plasma and fibrinogen concentrate (F + F) or fresh frozen plasma alone (F).

	F + F	F	*P*-value	severe-F + F	severe-F	*P*-value
Hb (g/dl)	7.08 ± 2.14	6.80 ± 1.92	>0.05	6.64 ± 2.60	6.45 ± 2.38	>0.05
PT%	44.2 ± 19.1	52.8 ± 17.5	0.0196	38.64 ± 17.5	40.78 ± 14.4	>0.05
Fibrinogen (mg/dl)	80.8 ± 21.8	83.6 ± 36.6	>0.05	80.2 ± 23.4	76.2 ± 39.8	>0.05
Estimated blood loss (ml)	4004 ± 2392	3284 ± 1862	>0.05	4596 ± 2641	3092 ± 2077	>0.05
RCC (unit)	20.5 ± 14.1	13.0 ± 8.07	<0.0013	29.5 ± 13.8	25.0 ± 4.85	>0.05
FFP (unit)	31.7 ± 18.8	30.0 ± 18.6	>0.05	40.2 ± 19.6	53.4 ± 18.5	0.0473
FFP/RCC	1.72 ± 0.76	2.48 ± 1.15	0.0002	1.40 ± 0.52	2.13 ± 0.63	0.0005

Sub-groups of the most severe patients were also compared; these required ≥18 red blood cell concentrate units.

Hb, haemoglobin concentration; PT%, prothrombin time activity percentage; RCC, red cell concentrate; FFP, fresh frozen plasma.

**Table 4:** Comparison of fibrinogen levels and other haemostatic parameters before treatment, and blood product usage in the groups of early-stage placental abruption requiring ≤10 units of red blood cell concentrate.

	Abruption F + F (n = 8)	Abruption F (n = 18)	*P*-value
Hb (g/dl)	7.27 ± 1.39	7.00 ± 1.93	>0.05
PT%	58.5 ± 16.6	59.5 ± 19.8	>0.05
Fibrinogen (mg/dl)	83.6 ± 20.9	82.5 ± 33.0	>0.05
Estimated blood loss (ml)	2504.6 ± 1306	2988.1 ± 1365	>0.05
RCC (unit)	7.00 ± 1.85	6.88 ± 2.19	>0.05
FFP (unit)	9.75 ± 2.91	19.0 ± 9.40	0.0122
FFP/RCC	1.47 ± 0.57	2.96 ± 1.60	0.0187

Hb, haemoglobin concentration; PT%, prothrombin time activity percentage; RCC, red cell concentrate; FFP, fresh frozen plasma; F + F, fresh frozen plasma and fibrinogen concentrate; F, fresh frozen plasma alone.
